# Excitability Changes in Intracortical Neural Circuits Induced by Differentially Controlled Walking Patterns

**DOI:** 10.1371/journal.pone.0117931

**Published:** 2015-02-17

**Authors:** Tomotaka Ito, Akio Tsubahara, Koichi Shinkoda, Yosuke Yoshimura, Kenichi Kobara, Hiroshi Osaka

**Affiliations:** 1 Department of Rehabilitation, Faculty of Health Science and Technology, Kawasaki University of Medical Welfare, Okayama, Japan; 2 Department of Biomechanics, Graduate School of Biomedical and Health Sciences, Hiroshima University, Hiroshima, Japan; Università di Trento, ITALY

## Abstract

Our previous single-pulse transcranial magnetic stimulation (TMS) study revealed that excitability in the motor cortex can be altered by conscious control of walking relative to less conscious normal walking. However, substantial elements and underlying mechanisms for inducing walking-related cortical plasticity are still unknown. Hence, in this study we aimed to examine the characteristics of electromyographic (EMG) recordings obtained during different walking conditions, namely, symmetrical walking (SW), asymmetrical walking 1 (AW1), and asymmetrical walking 2 (AW2), with left to right stance duration ratios of 1:1, 1:2, and 2:1, respectively. Furthermore, we investigated the influence of three types of walking control on subsequent changes in the intracortical neural circuits. Prior to each type of 7-min walking task, EMG analyses of the left tibialis anterior (TA) and soleus (SOL) muscles during walking were performed following approximately 3 min of preparative walking. Paired-pulse TMS was used to measure short-interval intracortical inhibition (SICI) and intracortical facilitation (ICF) in the left TA and SOL at baseline, immediately after the 7-min walking task, and 30 min post-task. EMG activity in the TA was significantly increased during AW1 and AW2 compared to during SW, whereas a significant difference in EMG activity of the SOL was observed only between AW1 and AW2. As for intracortical excitability, there was a significant alteration in SICI in the TA between SW and AW1, but not between SW and AW2. For the same amount of walking exercise, we found that the different methods used to control walking patterns induced different excitability changes in SICI. Our research shows that activation patterns associated with controlled leg muscles can alter post-exercise excitability in intracortical circuits. Therefore, how leg muscles are activated in a clinical setting could influence the outcome of walking in patients with stroke.

## Introduction

Recent progress in neuroimaging and transcranial magnetic stimulation (TMS) has made it possible to investigate cortical involvement during human walking and to identify the relationship between the control of walking and activity in the cerebral cortex [1–3]. Supraspinal centers likely play a greater role in the control of human walking than they do in the control of walking in quadrupedal mammals [4–7].

The cortex of the adult human brain has the potential to reorganize both functionally and structurally in response to physiological experiences (i.e., motor learning in healthy individuals) and pathological events (i.e., functional recovery in patients) [8, 9]. Walking is not an exception to this, and previous studies have revealed neuroplasticity in patients with stroke after several weeks of gait training [10–12]. However, these studies have focused on quantity of training [10–12], and the substantial elements required for generating walking-related cortical plasticity and its underlying neural mechanisms remain unknown.

Hornby et al. [13] have researched the effectiveness of qualitative characteristics of gait training. They clinically compared two different therapeutic strategies for walking and demonstrated that greater improvements in walking performance in ambulatory stroke survivors were observed in therapist-assisted training groups when compared to training groups that were robotic-assisted. However, it still remains unclear what qualitative elements in the assisted gait training facilitated the restoration of walking ability and the neuroplasticity necessary for gait improvement.

Recently, our study [14] using single-pulse TMS demonstrated a fundamental finding that conscious asymmetrical walking (AW), designed to explore the effect of intentional control over walking, produced both temporal and task-specific changes in motor-evoked potential (MEP) amplitudes compared to normal walking, which involves less conscious control. This result may suggest the importance of volitional drive in motor memory consolidation, even during gait training. However, the neurophysiological mechanism linking volitional walking control to motor memory consolidation is still unknown. Furthermore, although the AW task (which extends the duration of the left swing phase to activate the tibialis anterior (TA)) was adopted as an experimental model of conscious walking in our prior study [14], the effect of differentially controlled volitional walking (used to reinforce the duration of stance phase) on post-exercise cortical excitability remains unclear.

One advantage of paired-pulse TMS is its ability to assess excitatory and inhibitory mechanisms in primary motor cortex circuits, as expressed by short-interval intracortical inhibition (SICI) and intracortical facilitation (ICF). With regard to cortical excitability associated with motor learning, several studies using this paradigm have reported that excitability changes in intracortical neural circuits that control the muscle targeted for an individual task can be detected following active motor training [15–18]. In this view, it was hypothesized that different ways of volitionally controlling walking patterns would also induce different changes in the SICI and ICF. As shown in previous studies [15–18], we predicted that attenuation, or removal of an inhibitory effect, would be elicited by increased central command for activating leg muscles. This, in turn, might have a direct correlation with an increase in muscle activity necessary for accomplishing the various walking tasks. In the present study, three different walking conditions, including less conscious symmetrical walking (SW) and two qualitatively different types of conscious AW, were employed to prove these unresolved research questions. We believe that knowledge on the induced changes can further the understanding of elements critical for cortical reorganization or plasticity induced by walking.

## Materials and Methods

### Subjects

Eleven healthy male volunteers (age range: 19–22 years) without neurological or orthopedic disabilities in their lower extremities or trunks participated in this study. The purpose and methods of the study were clearly explained to all participants, and written informed consent was required prior to the experiment. The experiment was performed in conformity with the Declaration of Helsinki, and the experimental protocol was approved by the Research Ethics Committee of Kawasaki University of Medical Welfare.

### Walking conditions

Participants were instructed to walk on a treadmill (Sakai Medical Co. Ltd., Tokyo, Japan) at a speed of 2 km/h, and a metronome was used to set a tempo to each individual’s routine cadence. Based on the preset tempo, the following three walking conditions were determined according to our previous study [14].

(1) In SW, which was the control condition, the ratio of the left stance duration to the right stance duration was set at 1:1 (two beats for one gait cycle).
(2) In AW1, which had a prolonged swing phase duration on the left side, the ratio of the left stance duration to the right stance duration was set at 1:2 (three beats for one gait cycle).
(3) In AW2, which had a prolonged stance phase on the left side, the ratio of the left stance duration to the right stance duration was set at 2:1 (three beats for one gait cycle).

The three walking conditions were implemented on the treadmill with a walking speed of 2 km/h according to the metronome. Each walking condition consisted of a 7-min walking task. Walking paces were guided by the metronome, which was set to each individual’s routine cadence. Participants were required to practice the three different walking conditions prior to TMS examination, and no participant actually required additional practice time. In the current study, to give priority to excluding the influence of the amount of walking practice on the excitability changes in the intracortical circuits, the duration of preparative walking was restricted to the same amount across participants. Three min were given to participants to perform each walking condition on the treadmill without any instruction or support.

### General procedures

**Fig 1:**
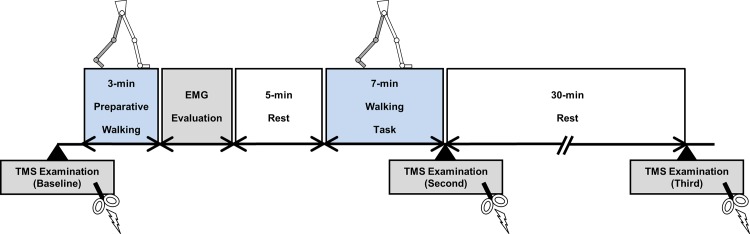
A schematic diagram of the experimental protocol. TMS examinations were conducted from a sitting position at three points during the experiment: before the commencement of 3-min preparative walking, immediately after the 7-min walking task, and 30 min post-task. EMG activity was recorded during the walking task in each condition following 3 min of preparative walking. The session of EMG analysis was followed by a 5-min rest, after which participants began the 7-min walking task.

A schematic diagram of the experimental protocol is illustrated in Fig. 1. Participants were instructed to sit on a chair and relax without making any muscle contractions. An initial TMS examination was then conducted to determine baseline values. After participants performed 3 min of preparative walking, electromyographic (EMG) activity was recorded during walking to clarify the characteristics of different walking patterns. Following a 5-min rest period, each participant performed a 7-min walking task during which no measurement or recording was performed. Further TMS examinations were performed in the sitting position immediately after the 7-min walking task and after a subsequent 30-min rest period in all experiments. Thus, TMS examinations were performed three times for each walking condition. The three walking conditions per individual were implemented on separate days with a randomized experimental sequence.

### EMG evaluation

After 3 min of preparative walking, surface EMG activity was recorded differentially from the left TA and soleus (SOL) using a telemetry electromyograph (MQ8; Kissei Comtec Co., Nagano, Japan) to disclose the characteristics of the individual walking conditions. Disposable silver/silver chloride surface electrodes with a 10-mm diameter (Blue Sensor P-00-S; Ambu Co., Ballerup, Denmark) were used for recording EMG activity during walking. Bipolar electrode pairs were placed longitudinally over the muscle belly at an interelectrode distance of 20 mm. A ground electrode was attached to the left lateral malleolus. Prior to electrode configuration, the skin was abraded with a skin preparation gel (Skin Pure; Nihon Kohden Co., Tokyo, Japan) and then cleaned with alcohol to reduce skin impedance. The EMG signals were sampled at 1000 Hz and stored on a personal computer for later off-line analysis. For standardization of the EMG signals, subjects were required to perform ankle dorsiflexion and plantar flexion for 5 s against the resistance given by the same examiner to measure the maximum voluntary contractions of the TA and SOL, respectively. To identify a gait cycle, pressure sensitive foot switches were placed under the left and right heels. A gait cycle was defined as the temporal interval between the beginning of heel contact to that of the next ipsilateral heel contact. After the completion of 3 min of preparative walking, 10 gait cycles were recorded under each condition.

Biological information analysis software (BIMUTAS II; Kissei Comtec Co., Nagano, Japan) was used to analyze the EMG data. After the recorded raw data were filtered using a frequency band of 20 Hz to 500 Hz, they were full-wave rectified and integrated. Subsequently, the integrated EMG (IEMG) during 10 gait cycles was divided by the required time, and the average IEMG per unit time was calculated. Relative value was obtained by dividing the IEMG of individual muscles by the peak amplitude in the maximum isometric contraction and was expressed as %IEMG.

### TMS procedures

TMS was delivered using a Bistim unit attached to two Magstim 200^2^ stimulators (Magstim Co., Dyfed, UK). A double-cone coil with an outer diameter of 110 mm was used to stimulate the leg area of the primary motor cortex. The coil was moved over the scalp to find an optimal location for eliciting activity in the left TA using the lowest stimulus intensity at baseline measurements, and was oriented so that the induced current flowed in the posterior-anterior direction in the brain. To ensure that the same location of the motor cortex was stimulated during each measurement, a swimming cap was tightly secured onto each participant’s head and fixed to the skin with surgical tape. The most suitable coil placement for activating the TA was marked on the cap. The TMS coil that was set on the head was removed during treadmill walking.

A paired conditioning-test technique [19], consisting of a subthreshold conditioning stimulus followed by a suprathreshold test stimulus, was used to measure SICI and ICF in the TA and SOL. These two measurement indices in the TA and SOL were simultaneously evaluated by stimulating a hot spot in the TA. Two magnetic stimuli were delivered to the right leg motor area via the same coil. The resting motor threshold (RMT) was determined while the TA muscle was at rest and was defined as the minimum stimulus intensity necessary for inducing an MEP with an amplitude of 100 μV in at least three out of five consecutive stimuli. The conditioning stimulus was set at 70% of the individual RMT so that it would not activate any corticospinal fibers and would not produce excitability changes in the spinal motor neurons [3, 20]. The test stimulus intensity was first set to 20% of the stimulator output above RMT. When the MEP amplitude in the TA was only detected at this stimulus intensity, it was then reset to 130% of the RMT to induce an MEP in the SOL as well. Stimulus intensity was expressed as a percentage of the maximum output of the stimulator. The timing, number, and order of stimuli were controlled with a pulse control device (Pulse Timer Unit; Medical Try System Co., Tokyo, Japan) and control software (Pulse Timer II; Medical Try System Co., Tokyo, Japan). The inter-stimulus intervals in the current study were set at 2.5 ms and 8 ms for SICI and ICF, respectively [17]. Ten trials were recorded for the SICI, ICF, and test stimulus alone, therefore, 30 trials were applied every 4 s in a pseudorandom order. EMG responses to the TMS were amplified using an 8-channel bio-amplifier (BA1008; TEAC Co., Tokyo, Japan), and stored in a personal computer. The sensitivity, time constant, and high-cutoff filter of the amplifier were set to 200 μV/0.5 V, 0.01 s, and 3 kHz, respectively. After obtaining three averaged MEPs, one each for SICI, ICF, and the test stimulus alone, the peak-to-peak amplitude was measured using examination software (Multi Stim Tracer; Medical Try System Co., Tokyo, Japan). The conditioned MEP amplitude was expressed as a percentage of the mean MEP amplitude obtained during delivery of the test stimulus alone. These data were displayed as ratios of either SICI or ICF.

### Statistical analyses

All statistical analyses were performed using IBM SPSS Statistics 20 (IBM SPSS Statistics Inc., Tokyo, Japan). One-way repeated measures analysis of variance (ANOVA) and Bonferroni post-hoc analysis were used to examine the differences in %IEMG in the two muscles (TA and SOL) under the three walking conditions (SW, AW1, and AW2).

In order to assess the effects of different walking patterns on intracortical circuits, changes from baseline in the SICI and ICF ratios following the walking tasks were analyzed using paired *t*-tests. SICI and ICF ratios in each walking task were divided by the baseline values (relative SICI ratio: R-SICI ratio; relative ICF ratio: R-ICF ratio, respectively) to compare excitability changes after the three walking conditions. Post-exercise intracortical excitabilities were compared immediately following the walking task and after the subsequent 30-min rest period using one-way repeated measures ANOVAs. When significant differences were found, multiple comparison tests were performed. Values of *P* < 0.05 were considered statistically significant.

## Results

### Characteristics of EMG parameters during the three walking conditions

**Fig 2:**
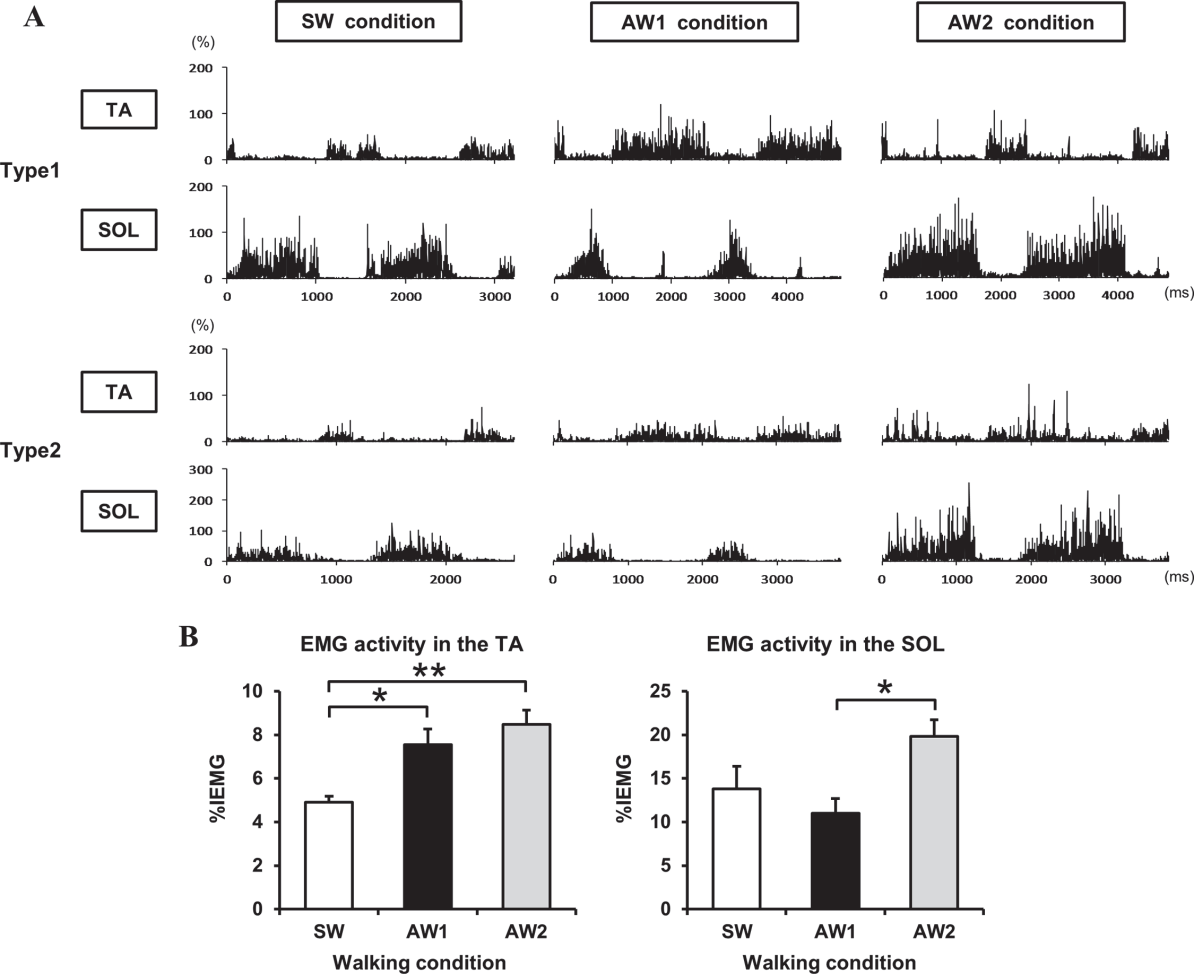
Characteristics of EMG activities during the three walking conditions (SW: symmetrical walking; AW1: asymmetrical walking 1; AW2: asymmetrical walking 2). (A) Two representative EMG types recorded from the left TA and SOL of two participants during the three walking conditions; Type 1 shows larger TA activity in the AW1 condition compared to the AW2 condition. In contrast, Type 2 shows larger TA activity in the AW2 condition. The vertical axis represents EMG activity in each muscle. All raw EMG data obtained during walking were divided by the averaged integrated EMG (IEMG) in the maximum isometric contraction and expressed as a percentage. The horizontal axis represents the duration of two gait cycles, starting from the initial left heel contact and ceasing at the third left heel contact. To clarify EMG activity patterns in each condition, the length of the horizontal axis in all figures is illustrated using equal length. (B) Comparisons of EMG activity in the TA and SOL during the three walking conditions. Relative value was obtained by dividing the IEMG of the individual muscles by the peak amplitude in the maximum isometric contraction and is expressed as %IEMG. Error bars indicate ± standard error. Significant differences among the three conditions are indicated by an asterisk (*P < 0.05; **P < 0.01).

Nine of the 11 subjects participated in the EMG evaluation during walking. Two types of representative EMG activity obtained during the three walking conditions are illustrated in Fig. 2A. One type showed larger TA activity in the AW1 condition compared to the AW2 condition, while the other type exhibited larger activity in the AW2 condition. As previously described, the tempo of an individual’s actual cadence was controlled by a metronome, which ranged from 67 to 95 steps per min during the SW condition and 44 to 63 steps per min during AW1 and AW2 conditions, respectively. Mean values and standard errors of the EMG activity are shown in Fig. 2B. A one-way repeated measures ANOVA revealed significant differences in the %IEMG of the left TA and SOL among the three walking conditions [*F*(2,16) = 8.758, *P* = 0.003; *F*(1.29,10.31) = 5.669, *P* = 0.032]. Compared to the SW condition, EMG activity in the TA was significantly augmented during AW1 (*P* = 0.027) and AW2 (*P* = 0.004) conditions. As for the SOL, a significant difference in the %IEMG was detected between AW1 and AW2 conditions (*P* = 0.020).

### Alterations in intracortical excitability following individual walking tasks

**Fig 3:**
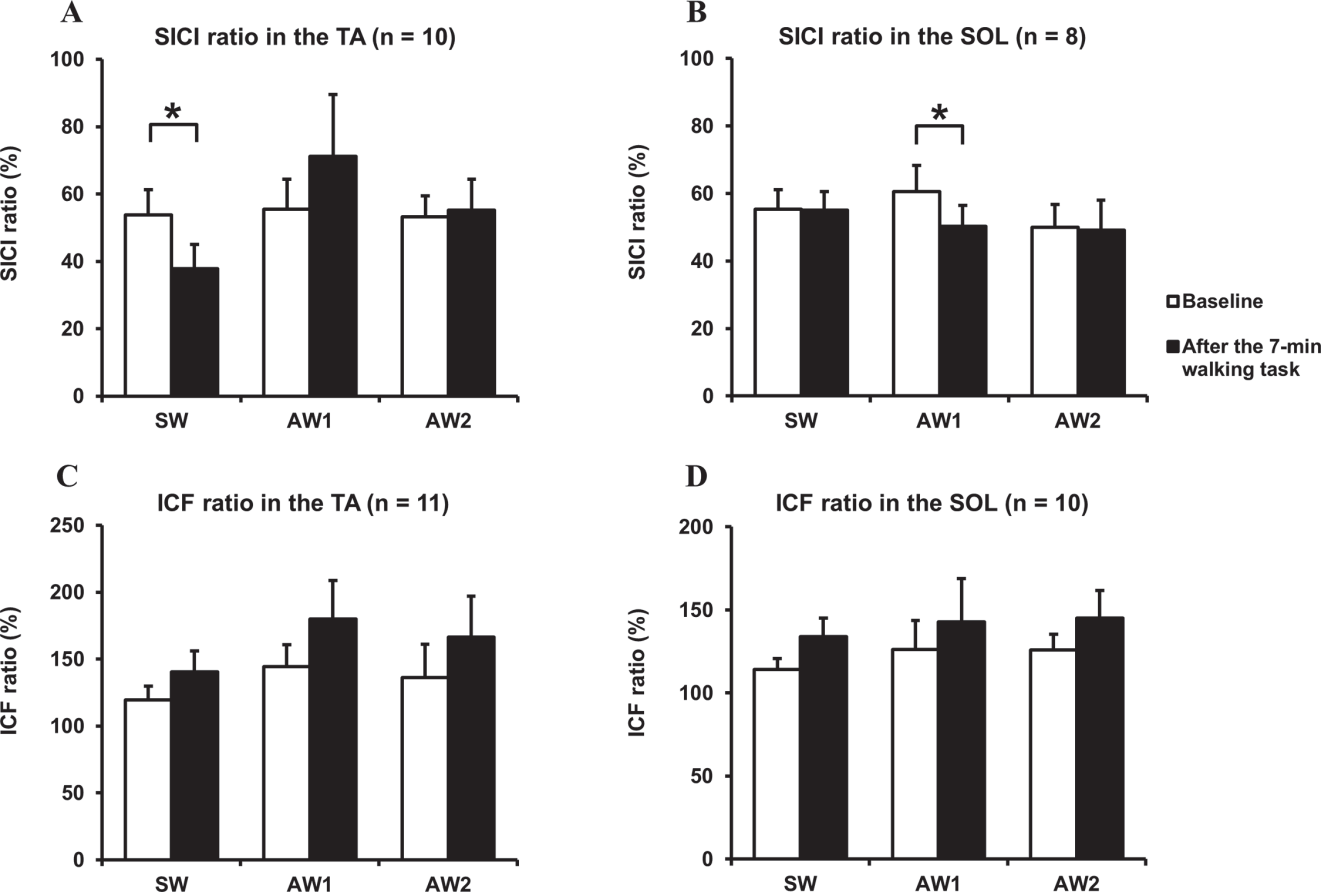
Changes in intracortical excitability in the TA and SOL following the three walking conditions (SW: symmetrical walking; AW1: asymmetrical walking 1; AW2: asymmetrical walking 2). Conditioned MEP amplitudes are presented as the percentage of the mean MEP amplitude during the test stimulation measured alone in the same recording session. All data are illustrated as short-interval intracortical inhibition (SICI) and intracortical facilitation (ICF) ratios. Error bars indicate ± standard error. (A) Excitability changes in the SICI ratio in the TA induced in 10 participants. (B) Excitability changes in the SICI ratio in the SOL induced in 8 participants. (C) Excitability changes in the ICF ratio in the TA induced in 11 participants. (D) Excitability changes in the ICF ratio in the SOL induced in 10 participants. Significant differences between baseline measurements and measurements taken after the 7-min walking task are indicated by an asterisk (*P* < 0.05).

Since one of the participants complained of fatigue during the experimental processes, he could not complete all of the walking conditions; however, this particular participant was able to complete the second TMS examination that took place following each walking task as well as the baseline examination. Mean values and standard errors of the SICI and ICF ratios in the TA and SOL are shown in Fig. 3. SICI could be evoked in 10 of the 11 participants in the TA, while SICI in the SOL was evoked in 8 of the 11 participants. Relative to the baseline measurements, the SICI ratio in the TA and SOL was observed to significantly decrease immediately after the walking task in the SW condition [*t*(9) = 3.054, *P* = 0.014] and in the AW1 condition [*t*(7) = 2.396, *P* = 0.048], respectively. The ICF in the TA could be evoked in all subjects, whereas the ICF in the SOL was evoked in 10 of the 11 subjects. No significant changes were observed in the ICF ratio, although it did tend to increase from the baseline level in the TA after the walking task in the AW1 condition [*t*(10) = 2.063, *P* = 0.066] and in the SOL after the walking task in both AW2 and SW conditions [*t*(9) = 2.020, *P* = 0.074; *t*(9) = 2.117, *P* = 0.063].

### Comparison of post-exercise excitability between the three walking conditions

**Fig 4:**
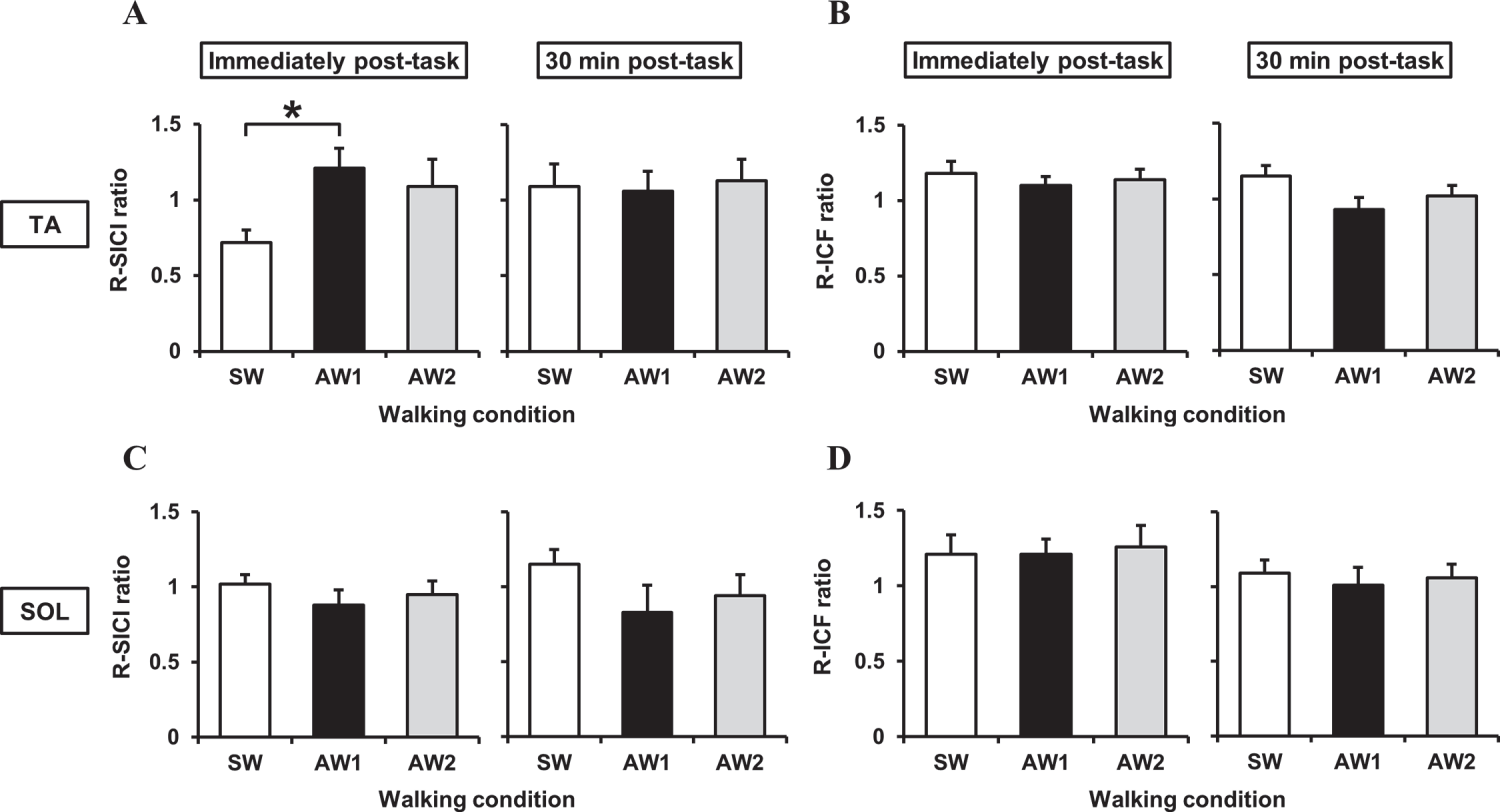
Comparisons of intracortical excitability following the three walking conditions (SW: symmetrical walking; AW1: asymmetrical walking 1; AW2: asymmetrical walking 2). The short-interval intracortical inhibition (SICI) and intracortical facilitation (ICF) ratios in each walking task were divided by the baseline values and are presented as R-SICI ratio and R-ICF ratio, respectively. Error bars indicate ± standard error. Upper section: Comparisons of R-SICI (A) and R-ICF (B) ratios in the TA immediately after the walking task (Left graph) and after the subsequent 30-min rest period (Right graph). Lower section: Comparisons of R-SICI (C) and R-ICF (D) ratios in the SOL immediately after the walking task (Left graph) and after the subsequent 30-min rest period (Right graph). Significant differences in each index among the three walking conditions are indicated by an asterisk (*P* < 0.05).

Fig. 4 presents the mean values and standard errors of the R-SICI and R-ICF ratios measured in both the TA and SOL immediately after the walking task and 30 min post-task for each condition. In the TA, there was a significant change in the R-SICI ratio [*F*(2,18) = 5.436, *P* = 0.014] between the SW and AW1 conditions only for measurements taken immediately after the walking tasks (*P* = 0.010). Therefore, the SW condition augmented the SICI as indicated by the decreased amplitude, whereas the SICI following the AW1 condition was likely diminished. However, no additionally significant differences in R-SICI and R-ICF ratios of the TA and SOL were detected among the other conditions in each measurement session.

## Discussion

In the current study, three walking conditions were employed to examine whether differentially controlled walking patterns could mediate excitability changes in the SICI and ICF of healthy volunteers. The results herein show that in the TA and SOL, there was a significant decrease in the SICI ratio after SW and AW1 conditions, respectively, which implied an increased inhibitory effect.

In humans, increased activity in the primary sensorimotor area, supplementary motor area, basal ganglia, and cerebellum has been found during walking using single-photon emission computed tomography [1]. Furthermore, near infrared spectroscopy has shown bilateral activity in the medial portion of the primary sensorimotor area and supplementary motor area during treadmill walking [2]. Nevertheless, most prior studies on short-term walking-induced cortical excitability have indicated no significant alterations in MEP amplitude between measurements performed before and after normal walking [14, 21–23]. Thus, while it is accepted that the motor cortex is involved in walking control in humans, cortical involvement during normal walking is not sufficient to elicit changes in post-exercise excitability in the primary motor cortex, which likely depend on the number of central commands controlling the legs [14].

With regard to the subsequent change in SICI after the SW condition, an increase in SICI in the TA was observed in the current study, which likely resulted from increased activity in gamma-aminobutyric acid (GABA)_A_-ergic cortical interneurons [24, 25]. Ziemann et al. [26] revealed that, in the presence of an ischemic nerve block, practice-dependent cortical plasticity was prevented or suppressed when subjects were pre-treated with lorazepam, a GABA_A_ receptor agonist. These findings suggested that a decrease in GABA-related cortical inhibition might facilitate practice-dependent plasticity in the human motor cortex, while an increase in this type of neurotransmission might depress practice-dependent plasticity. Since walking pattern control in the SW condition was relatively non-specific and since this is not an unusual task for healthy people to perform, it is speculated that the SW condition did not require motor learning processes that accompany practice-dependent plasticity in the primary motor cortex. Moreover, in terms of the characteristic difference between the three walking conditions, we found that an individual’s cadence in the SW condition tended to be increased relative to cadences in both AW conditions. As a consequence, the total number of steps taken in the SW condition was higher than in AW1 and AW2 conditions. This increased number of steps appears to provide participants with more opportunity to practice treadmill walking, which might influence the motor learning process and result in the reinforcement of SICI only in the SW condition.

The main purpose of this study was to explore potential factors involved motor learning-related cortical excitability following walking. To this aim, we adopted two different types of intentional walking patterns and focused on the control of extended swing and stance phases on the left side in AW1 and AW2 conditions, respectively. We hypothesized that an increase in central commands to the TA and SOL during walking would directly accompany changes in the SICI. However, our results did not completely support our hypothesis. As a result, a significant difference in the R-SICI ratio of the TA was detected only between SW and AW1 conditions immediately after walking tasks. Although the SICI ratio in the TA after the AW1 condition was not significant, there was an overall tendency for SICI to decrease when compared to the baseline level, which is in stark contrast to that after the SW condition. With respect to the changes in SICI elicited by short-term motor training, significant reductions in SICI have been demonstrated in leg muscles after repetitive skilled movement [17] and after active pedaling exercises [27]. The alteration of SICI in the TA after the AW1 condition appears to be consistent with changes shown in previous studies, which indicates that the volitional control of walking has the potential to mediate cortical reorganization.

It is well recognized that pedaling exercises involve reciprocal muscle activity in the lower limbs (activity that is similar to what is required for walking [28]), and that this activity is beneficial when retraining an individual to walk [29]. However, decreased SICI in the SOL was shown after active pedaling exercises in a prior study [27], while increased SICI in the SOL was detected after the AW1 condition in the present study. Potential reasons why these differences in SICI were observed during comparable tasks might be attributed to corticospinal excitability modulated by loading- [30] and walking-specific cortical involvement in each muscle [31, 32]. Load-related somatosensory inputs reportedly play an important role in facilitating corticospinal excitability in the TA during passive ground stepping, but not during passive air stepping [30]. Moreover, Capaday et al. [31] reported that MEPs evoked by TMS in the SOL were reduced during the stance phase of walking, while MEPs in the inactive TA during the stance phase were enhanced compared to the size of the MEPs elicited during voluntary ankle plantar flexion. This suggests that the corticospinal tract is less closely linked with the segmental motor circuits controlling the SOL than to those controlling the TA. Therefore, changes in the cortical excitability related to each muscle are assumed to be affected by the aforementioned factors. If this were the case, then this would explain why different changes in SICI were detected between walking and pedaling tasks despite the inclusion of voluntary and active elements in both movements.

Since the duration of the swing phase on the left side was prolonged in the AW1 condition, changes in the SICI observed following the AW1 condition were presumably induced by increased TA muscle activity, which begins in pre-swing and stops toward the end of the loading response [33]. It should be noted that more volitional gait control was required during the AW2 condition than during the SW condition. However, in contrast to the AW1 condition, which resulted in the significant augmentation of EMG activity in the TA, there was no significant difference in the R-SICI ratio between the SW and AW2 conditions. In terms of the EMG evaluation investigating the peculiarity of each walking condition, a significant difference in EMG activity of the SOL was only found between AW1 and AW2 conditions. Moreover, under the AW2 condition, EMG activity in both the TA and SOL during walking tended to increase relative to the SW condition. This means that muscle contractions in the TA and SOL were, more or less, simultaneously required. This was especially the case during the stance phase of the AW2 condition as the dual muscle contractions allowed participants to react to postural sway or to obtain stability with the extended duration of the left stance phase. Accordingly, as shown in the two types of representative EMG activity (Fig. 2), it is speculated that the increased TA activity during the AW2 task played a completely different role from that during the AW1 task. With regard to the alteration in SICI, we should also consider the influence of surround inhibition, which aids in the selective execution of desired movements and the suppression of non-target muscle groups [34]. Several previous studies have reported a reduced SICI in a hand muscle engaged in selective movement, whereas an enhanced or unchanged SICI was found in the untrained, inactive hand muscle during or following a selective movement [15, 35, 36]. Therefore, the mechanism underlying the different alterations in SICI in individual muscles under the AW1 condition might be related to surround inhibition, and this mechanism likely helps control extended TA activity observed in the swing phase.

As for changes in the ICF, the present study did not find any significant differences between measurements taken before and after walking in any of the three conditions. This result is in agreement with a previous study that described no significant changes in ICF after 20 min of active and passive robotic gait training using a driven gait orthosis [37]. Thus, condition-related changes in ICF were much less obvious than changes in the SICI in this study, which supports the idea that disinhibition is more important for intracortical plastic changes than an increase in ICF [15].

In conclusion, we revealed that different motor commands for controlling the leg muscles during walking elicited different changes in intracortical excitability. In other words, walking pattern-dependent cortical changes were generated by different ways of walking control under the same amount of walking exercise. Our novel findings demonstrated that both reinforcing motor commands (AW1, AW2 vs. SW) and altering how muscles are activated (AW1 vs. AW2) may be essential and crucial elements for inducing excitability changes in the SICI associated with motor learning. Thus, in the clinical setting, neuroplasticity should be considered even during walking because the activation pattern of leg muscles also influences intracortical neural circuits, implicating the improvement of motor function or walking ability in patients with stroke who suffer from gait disturbances. Owing to a technical difficulty in recording stable EMG activity for 7 min of a walking task, we could not evaluate the improvement of walking performance by means of measuring serial changes in EMG activity or variability of stance and swing duration. Since proficiency level or task difficulty of each walking task also mediates the motor learning process, further studies are needed to clarify the relationship between alterations in walking performance and cortical excitability.
